# Migrant Pathology Screening in the Pediatric Population: A Five-Year Retrospective Study From a Level II Hospital

**DOI:** 10.7759/cureus.53770

**Published:** 2024-02-07

**Authors:** Luísa Castello-Branco Ribeiro, Filipa Paixão, Francisca Costa, Paula Correia

**Affiliations:** 1 Pediatric Service, Child and Youth Department, Hospital Professor Doutor Fernando Fonseca, Amadora, PRT

**Keywords:** pediatrics, adolescent, child, screening, migrants

## Abstract

Introduction: The migrant population residing in Portugal has been growing. In 2015, the pediatrics department at Professor Doutor Fernando Fonseca Hospital, a level II hospital, implemented a screening for endemic pathologies in asymptomatic migrant children to enable their timely diagnosis and treatment. This study aimed to identify and characterize the main findings in the migrant pathology screening.

Methods: This was a retrospective and descriptive study of asymptomatic children and adolescents who underwent opportunistic screening for migrant pathology in a hospital setting between January 2016 and April 2021. Data analysis was performed using Microsoft Excel^®^.

Results: A total of 256 individuals were included in the study; 53.5% (137/256) were female, with a median age of eight years and two months (minimum five months; maximum 17 years and 10 months). The majority of the participants were from Guinea-Bissau (29.7%, 76/256), Angola (19.1%, 49/256) and Cape Verde (12.1%, 31/256) and had been residents in Portugal for a median time of five months (minimum two days; maximum three years and five months). A total of 42.6% (109/256) participants did not have the Portuguese vaccination schedule updated. Screening was carried out in an outpatient setting in 71.9% (184/256) of individuals. A total of 38.7% (99/256) presented screening alterations, including 65 anemia cases (18 caused by iron deficiency and one by sickle cell anemia), 5 cases of tuberculosis infection and 1 case of pulmonary tuberculosis, 1 of human immunodeficiency virus infection, 3 hepatitis B virus infection cases, 20 of parasitic infections and 2 cases of female genital mutilations.

Conclusion: The revised migrant pathology screening protocol enabled the detection of diseases with a significant impact on the health of individual children and adolescents. This protocol serves as a practical tool for accurately monitoring the health status of this population.

## Introduction

In 2021, for the sixth consecutive year, an increase was seen in the foreign population residing in Portugal [1]. In that year, there were 698,887 immigrants with a residence permit, the highest figure since the creation of the Portuguese Immigration and Border Service [1,2]. It is also known that, in Portugal, around 14% of foreigners are aged 19 or under and 9.4% are under the age of 15 years [1].

Data for 2021 shows that most immigrants are distributed around the country's coastal region, mainly in the district of Lisbon. Within the aggregate of immigrants currently residing in Portugal, 15.6% live in Lisbon, 6.1% in Sintra, 4.9% in Cascais and 3.4% in Amadora [2]. The immigrants living in the municipalities of Amadora and Sintra (our hospital's coverage area) come mainly from Brazil (26.9%) and the Portuguese-speaking African countries (in Portuguese, Países Africanos de Língua Oficial Portuguesa, or PALOP) (42.9%), particularly Cape Verde (19.5%), Guinea-Bissau (12%) and Angola (9%) [2].

The health of immigrant children and adolescents from resource-limited countries is a cause for concern, not only because of the wide range of pathologies (infectious, hematological and nutritional deficits) they suffer from, but also the burden of the economic and social deprivation that many of these families experience.

Given the substantial number of immigrants seeking healthcare at our hospital, many without prior medical follow-up in Portugal, our department (at Professor Doutor Fernando Fonseca Hospital) established a protocol in 2015 to guide migrant children and adolescents. This protocol guided, mainly, screening and management of the most common infectious and hematological diseases in resource-limited countries, to be applied opportunistically both in the outpatient clinic and the inpatient department. The screening is adapted to the country of origin and aims to diagnose pathologies at an early stage.

This study was presented as an Oral Communication at the 21st Portuguese National Congress of Pediatrics, Braga, Portugal, in October 2021 and received the Pierre Fabre Award - Portuguese Society of Pediatrics.

## Materials and methods

We designed a retrospective and descriptive study to describe the main pathologies of asymptomatic migrant children living in our hospital's coverage area. The study was carried out by analysing the clinical files of children and adolescents (between the ages of zero and 18 years) who were screened for migrant pathology at Professor Doutor Fernando Fonseca Hospital (a level II district hospital in the Lisbon metropolitan area) between January 2016 and April 2021.

We defined eligible for screening migrant as any asymptomatic child or adolescent who was born in and/or migrated from a resource-limited country, considering that this population has a low rate of medical follow-up in their country of origin, thus posing a higher risk of undiagnosed illnesses.

Taking into account that our hospital does not have a specific consultation for migrants, we used a non-random convenience sampling method based on patients who underwent a (1) human immunodeficiency virus (HIV)-1/2 test and (2) fecal examination for parasites. All symptomatic patients at the time of screening were excluded.

The protocol is individually tailored to each child, in accordance with the attending physician overseeing the patient. Globally, it includes screening for hematological diseases (complete blood count with reticulocytes and hemoglobin electrophoresis) and screening for infectious diseases such as tuberculosis (tuberculin test and/or interferon gamma release assay, or IGRA, and/or chest radiograph), syphilis (Venereal Disease Research Laboratory, or VDRL), hepatitis B virus (HBV) infection (hepatitis B surface antigen, or HBsAg; antibody to hepatitis B core antigen, or anti-HBc; antibody to hepatitis B surface antigen, or anti-HBs), hepatitis C virus (HCV) infection (antibodies to hepatitis C virus, or anti-HCV), hepatitis A virus (HAV) infection (antibodies to hepatitis A virus, or anti-HAV), HIV infection (antibodies to HIV-1/2, fourth-generation antigen/antibody combination HIV-1/2) and intestinal parasite infection (fecal examination for parasites).

Our protocol also suggests screening for other sexually transmitted diseases (polymerase chain reaction, or PCR, for *Neisseria gonorrhoeae* and *Chlamydia trachomatis*)* *if there is a history of sexual abuse, unprotected sex, or abnormal vaginal or urethral discharge. We also recommend performing *Trypanosoma cruzi *serology in children who have migrated from endemic regions (Central and South America) and *Toxocara* spp. serology in children who migrated from South America and Southeast Asia. We search for *Schistosoma* spp. infection by serology if the child migrated from Sub-Saharan Africa, Southeast Asia and some areas of Latin America (Venezuela, Brazil and the Caribbean); if there is a history of hematuria, we recommend performing a parasitological examination of urine, searching for *Schistosoma haematobium*. If there is a history of weight loss or diarrhea, our protocol suggests searching for the *Giardia lamblia* antigen in the stool. In children with a vegetarian/vegan diet or excessive milk intake and in female adolescents with heavy menstrual bleeding, we investigate iron deficiency (through ferritin dosing). We also recommend, in vegetarian or vegan patients, dosing vitamin B12 and folate. Our protocol also recommends screening female genital mutilation (FGM) in girls and female adolescents who have migrated from Guinea-Bissau, Senegal and Guinea-Conakry.

We define a migrant with abnormal screening as any child or adolescent with abnormalities in the screening, such as anemia, hemoglobinopathy, latent or active tuberculosis, syphilis, acute or chronic hepatitis B, hepatitis C, hepatitis A, HIV infection, intestinal parasitosis, or any other disease documented through analytical screening (infection with *N. gonorrhoeae*, *C. trachomatis*, *T. cruzi,* *Schistosoma* spp., or nutritional deficiencies such as iron deficiency, vitamin B12 deficiency or folate deficiency). Young adolescents who have undergone female genital mutilation are also included in those with abnormal screening.

We analyzed sociodemographic (sex, age, country of birth and of migration, date of migration and migration route), clinical variables (body mass index, or BMI, and vaccination status at the date of screening) and laboratory variables (results of the migrant screening). We also looked for children who had been referred to the Social Service by the doctor who performed the screening and the reason for the referral. For patients with an abnormal screening, we analyzed subsequent outpatient follow-up, the treatment given and the consultation drop-out rate.

For the statistical analysis, we used Microsoft Excel® (Microsoft Corporation, Redmond, WA) to calculate the median, minimum and maximum values for variables with a non-normal distribution.

## Results

Demographic characterization

A total of 256 children and adolescents were included; 53.5% (137/256) were female, with a median age of eight years and two months (minimum five months; maximum 17 years and 10 months). They had been in Portugal for a median of five months (minimum two days; maximum three year and five months). The demographic characterization of the individuals included in the study and of those with abnormal screening is explicitly stated in Table 1.

**Table 1:** Demographic characterization of the individuals included in the study and those with an abnormal screening n = absolute number

	Sample (n=256)	Individuals with screening alterations (n=99)
Sex		
Female, n (%)	137 (53.5)	52 (52.5)
Male, n (%)	119 (46.5)	47 (47.5)
Age (months), median (minimum, maximum)	62 (5, 214)	73 (6, 212)
Place of birth		
Guinea-Bissau, n (%)	76 (29.7)	36 (36.4)
Angola, n (%)	49 (19.1)	23 (23.2)
Cape Verde, n (%)	31 (12.1)	9 (9.1)
Brazil, n (%)	30 (11.7)	5 (5.1)
Others, n (%)	70 (27.3)	26 (26.3)
Municipality of residence		
Sintra, n (%)	138 (53.9)	49 (49.5)
Amadora, n (%)	108 (42.2)	44 (44.4)
Other, n (%)	10 (3.9)	6 (6.1)

**Figure 1:**
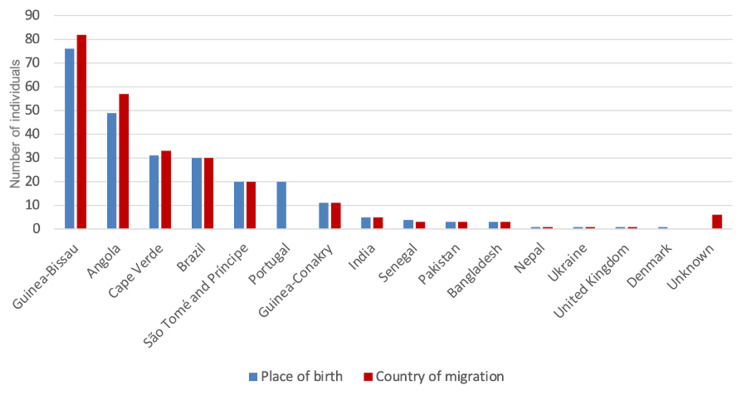
Place of birth and country of migration of the individuals included in the study

A total of 68.8% (176/256) individuals were born in PALOP countries, Guinea-Bissau (29.7%, 76/256), Angola (19.1%, 49/256) and Cape Verde (12.1%, 31/256), and 11.7% (30/256) were from Brazil. It is worth noting that 7.8% (20/256) of the individuals included in the study were born in Portugal but migrated or lived for long periods in resource-limited countries before returning to Portugal (Figure 1). Regarding the country of migration, the majority of the children and adolescents in the study migrated from Guinea-Bissau (32%, 82/256), Angola (22.3%, 57/256) and Cape Verde (12.9%, 33/256).

We highlight three cases that were included in the study: a child who was born in the United Kingdom but migrated to Angola and from there to Portugal; a child who was born in Denmark but migrated to India and from there to Portugal; and a child who was born in São Tomé and Príncipe, migrated to the United Kingdom and then to Portugal.

Characterization of health surveillance

A total of 48% (123/256) of the individuals had all the vaccines recognized in the Portuguese vaccination schedule, and in 9.4% (24/256), the vaccination status was unknown. In total, 80.5% (206/256) had a normal BMI, 7.4% (19/256) were thin (BMI <P3), 3.1% (8/256) were overweight (P85<BMI<P97) and 6.6% (17/256) were obese (BMI >P97). The BMI of 2.3% (6/256) at the time of screening was unknown.

A total of 71.9% (184/256) of our sample were screened at the outpatient clinic and 28.1% (72/256) were screened at the inpatient department. Among those screened at the outpatient clinic (n=184), 63.6% (117/184) were referred by the emergency department, 12% (22/184) by the primary healthcare center, 10.3% (19/184) by other hospital specialties/subspecialties and 8.7% (16/184) by inpatient care. In 4.9% (9/184), the origin of the referral to the outpatient clinic was unknown.

During the screening, 8.2% (21/256) of the children and adolescents were referred to the social services. Referral reasons included economic difficulties (47.6%, 10/21), multiple absences from appointments (14.3%, 3/21), bureaucratic difficulties in the legalization process (14.3%, 3/21), FGM (9.5%, 2/21), history of multiple unexplained accidents (4.8%, 1/21), physical abuse (4.8%, 1/21) and illegal abortion (4.8%, 1/21).

Characterization of individuals with an abnormal screening

An abnormal screening was found in 38.7% (99/256) of the children and adolescents (Table 2).

**Table 2:** Findings in the pathology screening of migrants HBV: hepatitis B virus; HIV: human immunodeficiency virus

	Number of screenings	Number of individuals with an abnormal screening	% of individuals with an abnormal screening
Anemia	216	65	30.1
Normocytic anemia		27	12.5
Microcytic anemia		38	17.6
Hemoglobinopathies	171	30	17.5
Sickle cell trait		25	14.6
Sickle cell disease		1	0.6
Other hemoglobinopathies		4	2.3
Intestinal parasitosis	241	19	7.9
Strongyloides stercoralis		8	3.3
Giardia lamblia		8	3.3
*Toxocara* spp.		2	0.8
Ascaris lumbricoides		1	0.4
Trichuris trichiura		1	0.4
Tuberculosis	180	6	3.3
Tuberculosis infection		5	2.8
Tuberculosis disease		1	0.6
HBV infection	239	3	1.3
Nonreplicative chronic HBV infection		2	0.8
Replicative chronic HBV infection		1	0.4
HIV infection	246	1	0.4
Female genital mutilation	12	2	16.7

**Figure 2:**
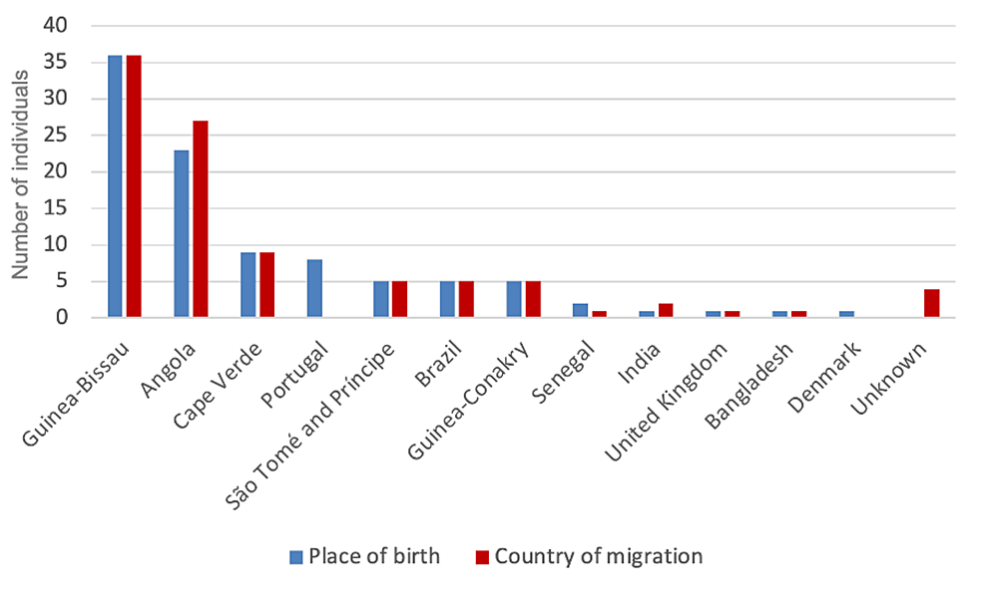
Place of birth and country of migration of individuals with an abnormal screening

Regarding the place of birth, 73.7% (73/99) were from the PALOP, with a significant contribution from Guinea-Bissau (36.4%, 36/99) and Angola (23.2%, 23/99) (Figure 2).

Hematological Diseases

Complete blood count was carried out on 84.4% (216/256) individuals, 30.1% (65/216) of whom had anemia: 58.5% (38/65) microcytic and 41.5% (27/65) normocytic. Iron deficiency anemia was diagnosed in 27.7% (18/65) of the patients, whose median age was six years and three months (minimum one year and one month; maximum 16 years and 8 months).

A total of 66.8% (171/256) patients underwent hemoglobin electrophoresis: 25 had sickle cell trait and one teenager was diagnosed with sickle cell anemia. This 15-year-old adolescent was a native of Guinea-Conakry and had no recent history of painful complaints. When questioned again (after the results), she mentioned having had some previous episodes of limb pain that responded to oral analgesia. There were also two cases of beta-thalassaemia minor, one child with AC haemoglobinopathy, one case of alpha-thalassaemia and two probable cases of alpha-thalassaemia, both awaiting genetic study.

Infectious Diseases

Concerning parasite screening, 93% (238/256) patients underwent fecal examination for parasites, 16.8% (43/256) underwent serology for *Strongyloides stercoralis* and 12.9% (3/256) underwent serology for *Toxocara* spp. A total of 20 intestinal parasites were diagnosed in 19 patients: *G. lamblia* (42.1%, 8/19), *S. stercoralis* (42.1%, 8/19), *Toxocara* spp. (10.5%, 2/19), *Trichuris trichiura* (5.3%, 1/19) and *Ascaris lumbricoides* (5.3%, 1/19). One patient had a co-infection with two intestinal parasites (*S. stercoralis* and *Toxocara* spp.). A total of 26.3% (5/19) had eosinophilia (>500 cells/μL): four with *S. stercoralis* infection and one with *A. lumbricoides.* All of them had mild to moderate eosinophilia, with no cases of hypereosinophilia (>1500 cells/μL). A total of 84.2% (16/19) had a normal BMI; 5.3% (1/19) were thin and 5.3% (1/19) were overweight. As for the concomitant presence of anemia, 2.6% (5/19) had microcytic anemia and 2.1% (4/19) had normocytic anemia. All patients were treated.

Tuberculosis screening was carried out on 70.3% (180/256) of the patients: 66.0% (169/256) underwent IGRA, 7.4% (19/256) underwent a tuberculin test and 3.1% (8/256) underwent both screening tests. Of the 180 patients screened for tuberculosis, 3.3% (6) showed alterations: five cases of tuberculosis infection and one case of pulmonary tuberculosis with endobronchial involvement.

Immunization status against HBV was investigated on 93.4% (239/256) patients, of whom 63.6% (152/239) were immune, 35.1% (84/239) were non-immune, two patients had chronic nonreplicative infection (from Guinea-Conakry and Cape Verde, diagnosed at 7 years and 10 months and 16 years and 3 months, respectively) and one had chronic replicative infection (aged 17 years and 5 months, from Guinea-Bissau). Of the 84 non-immune patients, 48.8% (41/84) had not been vaccinated against HBV.

Anti-HAV testing was performed on 43.4% (111/256) individuals: 55% (61/111) were immune (although the vaccination status of these children against HAV was unknown) while 45% (50/111) tested negative.

HCV infection was screened in 91% (233/256) individuals and syphilis in 59.8% (153/256), including 28.1% (43/153) adolescents aged 13 or over, and no cases were diagnosed.

A total of 96.1% (246/256) were screened for HIV. One HIV-1 infection (vertical transmission) case, a native of Guinea-Bissau, was detected at 16 months. Maternal diagnosis of HIV-1 infection was made at the same time. 

Social Risk

The population sample included 50 (19.5%) female individuals from Guinea-Bissau, Senegal and Guinea-Conakry. FGM was sought in 24% (12/50) of these girls, among whom two girls with FGM were identified, both from Guinea-Bissau (11 years and 5 months; 17 years and 3 months). In the remaining individuals with an indication for screening (76%, 38/50), there was no clinical record of genital observation.

Follow-Up

Of the 99 individuals with an abnormal screening, 87.9% (87/99) had a follow-up related to the finding at our hospital, with the occasional need for follow-ups in more than one consultation. Most patients were followed up in general pediatrics (59.8%, 52/87), pediatric infectious diseases (34.5%, 30/87) and pediatric hematology (14.9%, 13/87). Among the patients followed up in the consultation, 17.2% (15/87) were still being followed up at the time of data collection, 39.1% (34/87) were discharged after medical advice on their pathology and 43.7% (38/87) abandoned the consultation. Among those who were discharged, the median follow-up time was eight months (minimum 15 days, maximum two years and six months).

## Discussion

Our hospital's protocol for migrant children differs from other similar protocols (Table 3), all applied to children from resource-limited settings or from low socioeconomic backgrounds.

**Table 3:** Comparison of laboratory studies recommended in some migrant pathology screening protocols HCG: human chorionic gonadotropin; HIV: human immunodeficiency virus; IGRA: interferon gamma release assay; RF: risk factors; VDRL: Venereal Disease Research Laboratory

	Professor Doutor Fernando Fonseca Hospital	American Academy of Pediatrics [3]	The Royal Children's Hospital Melbourne (screening protocol for refugees) [4]	Wake Forest School of Medicine and Brenner Children’s Hospital [5]	European Journal of Pediatrics [6]	Pediatric Clinics of North America [7]
Complete blood count	x (with reticulocytes count)	x	x		x	x
Hemoglobin electrophoresis	x	RF				
Tuberculosis (tuberculin test and/or IGRA)	x	x	X	All (2-14 years old), RF (under two years old), chest radiograph (older than 14 years)	Everyone under five years old (above five years, if RF)	All (2-14 years old), RF (under two years old), chest radiograph (older than 14 years)
Syphilis (VDRL)	x	x	RF	RF	RF	x
Hepatitis A virus (anti-HAV)	x			X		
Hepatitis B virus (HBsAg, anti-HBs and anti-HBc)	x	x (just HBsAg)	X	X	x	x
Hepatitis C virus (anti-HCV)	x	RF	RF	X	RF	RF
HIV	x	x	RF	X	RF	Under 13 years, if unknown perinatal exposition; 13 years or more in everyone
Intestinal parasites	x	x	X	X	RF	x
Ferritin			X		If anemia	
Sexually transmitted diseases (PCR *Neisseria gonorrhoeae* and *Chlamydia trachomatis*)	RF	RF	RF		RF	
Lead		x				Under six years
Depending on specific RF	*Trypanosoma cruzi*, *Giardia lamblia*, *Schistosoma* spp., *Toxocara* spp., vitamin B12, folate	HCG, thyroid-stimulating hormone, glucose-6-phosphate dehydrogenase deficiency, *Schistosoma *spp., malaria	Calcium, alkaline phosphatase, phosphorus,*Schistosoma* spp., malaria, varicella-zoster virus, *Helicobacter pylori*, vitamin deficiencies	*Schistosoma* spp., *Strongyloides* spp., *Toxocara canis*, lymphatic filariasis, chest radiograph	*Schistosoma* spp., *Strongyloides* spp., vitamin D, malaria	Malaria, *Trypanosoma cruzi*, *Schistosoma* spp., *Strongyloides* spp.

Overall results

In our study, most individuals were from the PALOP, which reflects the origin of the migrants living in Amadora and Sintra. Notably, 42.6% (109/256) of the children had not received updated immunizations consistent with the Portuguese vaccination schedule recommendations. This may be due to the difficulty in accessing health care (especially in 2020 and 2021, due to the COVID-19 pandemic) associated with the fact that, on average, the individuals in the study had only been in Portugal for five months at the time of the screening.

Findings

Several treatable pathologies, with an important long-term impact on the health of these individuals, were found.

Anemia is a public health concern in both developed and resource-limited countries [8]. Overall, in 2010, anemia affected 32.9% of the population [9]. We highlight the different prevalence rates between Europe and Africa in the preschool age (26.5%/74.6%) and school age (9.3%/13.2%), respectively [8]. Iron deficiency is the main cause of anemia in children worldwide, and its incidence is very high in children up to the age of three, decreasing in older children [10]. In our study, 30.1% (65/216) of the screened individuals were diagnosed with anemia, of which 27.7% (18/65) had iron deficiency. The median age of patients with iron deficiency anemia (six years and three months) was higher than usual, suggesting that anemia is not associated with breastfeeding or menstrual loss, but with nutritional deficiencies characteristic of resource-limited countries. One of the main differences in our protocol when compared to others (Table 3) is the inclusion of hemoglobin electrophoresis. In February 2022, screening for sickle cell disease was included in the Portuguese National Neonatal Screening Program (pilot study) due to the importance of the disease in the Portuguese population. We therefore believe it is extremely important to systematically include this test in our protocol, since the population to which it applies often originates from African countries, where hemoglobinopathies are common. An adolescent was diagnosed with sickle cell anemia. A timely diagnosis holds great importance, since a proper medical follow-up allows for the reduction of morbidity and mortality associated with the pathology, namely, by carrying out the recommended vaccination schedule for these patients and by screening for cardiac, neurological and nephrological complications. We also emphasize the significance of having identified 25 cases of sickle cell trait, which, while not posing an immediate risk to the child, can have future consequences, especially for conception counseling.

In our study, we identified 19 children with asymptomatic intestinal parasitosis (7.8%, 19/241). Eight of these children were infected with *G. lamblia* (3.3%, 8/241), a figure that was within expectations, given that asymptomatic infections with this agent affect between 3% and 7% of healthy children [11]. Intestinal parasitosis is an infection that is more frequent in resource-limited countries where hygiene care is more precarious [12], with the highest infection rate in sub-Saharan Africa, Asia and Latin America [13]. In Portugal, studies on the prevalence of intestinal parasitosis are scarce, but it is thought that there has been a significant reduction in its prevalence, associated with better hygiene care [13,14]. The most frequent parasites in Portugal are *G. lamblia* and *Enterobius vermicularis*, with the remaining helminths rarely identified [12]. Our study did not identify any cases of parasitosis caused by *E. vermicularis* probably because the Graham technique was not used. The absence of eosinophilia in patients with intestinal parasitosis is common in patients with chronic exposure to parasites, so it is unsurprising that only 26.3% (5/19) of patients diagnosed with intestinal parasitosis displayed eosinophilia [15]. However, it is established that patients with eosinophilia despite a negative fecal examination for parasites may still be in an early stage of parasitic infection, making it reasonable to further investigate the possibility of parasitic infection if typical symptoms are present [15].

It is estimated that more than 1.7 billion people worldwide are infected with *Mycobacterium tuberculosis* [16], with a higher prevalence in South-East Asia, Africa and the Western Pacific [17]. According to the World Health Organization (WHO), 11% of the new cases of tuberculosis diagnosed in 2018 were diagnosed in children under the age of 15 and 13.8% of deaths from tuberculosis occurred in children at this, suggesting a higher mortality rate in this age group [17]. The aim of screening programs is to identify infection and prevent the development of disease and its transmission. Pediatric patients, particularly those under the age of five, have a higher risk of progression from tuberculosis infection to disease when compared to adults [18]. On the other hand, children between the ages of 5 and 10 are more often asymptomatic and are a reservoir for the reactivation of the infection in adolescence. It is therefore essential to identify these children to control the disease worldwide. Some international protocols only recommend systematic investigation in children under five years of age (if they have migrated from a country where they were exposed to unfavourable conditions such as forms of violence, socioeconomic deprivation and limited access to heath care [6]) due to symptom non-specificity and the severity and progression of the disease in this age group [19] (Table 3), extending screening to older children with risk factors such as migration from a country with a high endemicity for tuberculosis. The migrant screening applied at our hospital allowed us to diagnose five cases of tuberculosis infection (with three of them over the age of five and none with a known family history of tuberculosis) and one case of pulmonary tuberculosis with endobronchial dissemination (a male child, aged 7 years and 10 months, from Guinea-Conakry who underwent IGRA). Subsequently, respiratory symptoms were detected, which led to hospitalization with a positive PCR for *M. tuberculosis* in the sputum (sensitive to first-line antibacillars), and antibacillary therapy was started. Thus, in our screening, 66.7% (4/6) children with *M. tuberculosis* infection were older than five years.

Infection with HBV can cause acute hepatitis and liver failure and develop into chronic hepatitis with the possibility of triggering cirrhosis and liver cancer [20]. Chronic HBV infection has a higher prevalence in Asia, Africa, Eastern Europe and Latin America where the main route of transmission of the disease is vertical [20,21]. Most children with chronic HBV infection are asymptomatic [20]. In our study, 48.8% (41/84) of those with negative anti-HBs had up-to-date immunizations in accordance with the Portuguese vaccination schedule. However, it is known that the HBV vaccine is extremely effective at preventing HBV infection and that, although the levels of antibodies induced by the vaccine tend to decline over time, its effectiveness remains high for more than 30 years after immunization [22]. The Centers for Disease Control and Prevention (CDC) does not generally recommend confirming immunity in immunocompetent patients vaccinated with an adequate HBV vaccination schedule [22]. CDC and the Portuguese vaccination schedule recommendations for post-vaccination serology include children of HBsAg-positive mothers, healthcare workers, dialysis patients and HIV-infected patients [22,23]. The Portuguese vaccination schedule also recommends that post-vaccination serology should be carried out on transplanted patients and candidates for solid organ transplants [23], and CDC recommends testing in the case of other immunocompromised patients and patients whose sexual partners are HBsAg-positive [22]. We believe that screening for HBV infection should always include HBsAg, but that the other serological markers (anti-HBs and anti-HBc) should probably only be included in the event of risk factors, namely those described above, in the absence of an adequate vaccination schedule or in the absence of a credible family/past history. Considering current recommendations, the study on HBV infection in patients with confirmed immunization in vaccination records should be reconsidered.

No cases of HCV infection were detected in our study. The relevance of screening for this pathology remains controversial given the scarcity of prevalence data worldwide in migrant children [6].

HAV infection is the most common form of acute viral hepatitis worldwide, and is endemic in resource-limited countries with poor hygiene and sanitation conditions, especially most countries in Africa, Asia and Central and South America, where most people get infected during childhood [24]. Most international screening protocols for migrant pathology do not include screening for HAV infection (Table 3). However, we consider pertinent to include screening for this pathology in migrant children and adolescents, since there is an indication for vaccinating non-immune children who will be traveling to HAV endemic countries, as is often the case in this population [25]. It should also be noted that the American Academy of Pediatrics and the CDC recommend routine vaccination against HAV for all children over the age of one who have no evidence of immunity [5]. Although no cases of acute HAV were detected in our study, 45% (50/111) had no immunity for the infection.

Most children infected with HIV reside in resource-limited countries with 90% living in sub-Saharan Africa, with most of them infected through vertical transmission [26]. According to UNICEF, 2.58 million children and young people between the ages of zero and 19 were infected with HIV in 2020, with around 740 new infections every day in this age group [27]. In the absence of treatment, most children with HIV infection progress to acquired human immunodeficiency syndrome and die before the age of five [28]. Treatment with antiretroviral drugs makes it possible to change the natural history of the disease, with a significant reduction in morbidity and mortality. This explains the need to screen migrants from highly endemic countries for HIV infection. One case of HIV-1 infection (vertical transmission) was diagnosed leading to follow-up and treatment.

In our study, we did not identify any *Treponema pallidum* infections. Although several protocols recommend investigating syphilis only in the presence of risk factors (Table 3), such as suspected sexual abuse or congenital infection, it is known that the proportion of asymptomatic children is significant and that screening costs are relatively low [6]. Besides that, the prevalence of sexual abuse is higher in the migrant pediatric population, which is probably underreported, which may justify universal screening [6]. Other protocols recommend universal screening due to the frequent absence of gestational medical records and gestational screening practices [7]. More studies on the prevalence of these pathologies in migrant children should be carried out to draw conclusions.

FGM concerns any procedure that involves the partial or total removal of a woman's external genital organs or causes injury to them for non-medical reasons, with numerous complications [29]. WHO estimates that 100 to 140 million women worldwide have undergone FGM and that three million girls are at risk of undergoing this practice every year in the African continent [29]. This practice is carried out in many African countries (Guinea-Bissau has an estimated prevalence of FGM of 45% [30]) and some countries in Asia and Middle East [29]. Globalization and migratory pressure have increased the number of female children and adolescents living abroad who are at risk of or have undergone FGM [29]. Therefore, and taking into account the presence of immigrants in Amadora and Sintra from countries where FGM is common, it is necessary to pay special attention to these children, not forgetting the possibility of FGM during school vacations when visiting the countries of origin. Two cases of FGM were found in the sample of 50 female children with screening indication. However, in 76% (38/50) children, there was no record of genital observation. This highlights the importance of carrying out a systematic objective examination of all children who are seen, whether in hospitalization or in consultation, in order to detect possible alterations, which are often underdiagnosed.

We also highlight the problem of appointment drop-out rate. Among the individuals with an abnormal screening and who were followed up by a hospital doctor, 43.7% (38/87) abandoned the consultation, which is a very high figure considering the harmful effect that the worsening of some of these pathologies could have on the health of these individuals.

We also emphasize the need for gathering a suitable and comprehensive social history to assess the need for evaluation by Social Services, as was done in 8.2% (21/256) of children and adolescents.

To our knowledge, this study is the first to implement a screening protocol for endemic pathologies in resource-limited countries for the immigrant population in Portugal. We do not know what impact the implementation of a screening protocol in primary health care would have, but it is possible that important pathologies would be detected earlier, covering a greater number of migrant children and adolescents and possibly helping public health strategies.

Limitations

First, the sample was selected in an opportunistic context and may be under-representative of the immigrant population in the municipalities of Amadora and Sintra; therefore, the results cannot be extrapolated to the entire population. Besides, as this is a single-center observational study, it may be under-representative of the Portuguese immigrant population. We believe that the implementation of this protocol at the primary healthcare level or in a multicenter study could yield interesting results with essential contributions to the public and individual health of these children and adolescents. Second, the study was retrospective and some patients' medical files were incomplete. We recommend the inclusion of all relevant clinical data, as well as the documentation of a comprehensive physical examination for all children and adolescents in the clinical file.

We also point toward a relevant selection bias since the sample was selected by looking for patients who had both HIV serology tests and fecal examinations for parasites in their medical file. Those patients who had undergone the migrant screening protocol but, for any reason, did not have either of these two parameters were not included in the study.

At last, as previously mentioned, the protocol is adapted to each child and adolescent, taking into account the country of origin, risk factors, clinical history and clinical examination. It is therefore up to each doctor to choose which variables to analyze in each child. For this reason, in some cases, children may not have been screened as thoroughly as they could have been and the data collected in this work may be more difficult to interpret. To overcome this limitation, we suggest the creation of a specific consultation for screening migrant-related pathologies or the development of a support checklist (but not mandatory) for the attending physician conducting this screening.

## Conclusions

Our hospital's recently updated migrant pathology screening protocol allowed the diagnosis of diseases that have a significant impact on the individual health of these children and adolescents. Hematological pathologies proved to be the most commonly encountered. This protocol intends to be a working tool for correctly monitoring the health status of this population. We emphasize the need for a careful anamnesis and a complete clinical examination of all migrants.

Further studies are needed to investigate the prevalence of pathologies in the pediatric migrant population currently residing outside our coverage area. These studies could serve as a basis for the implementation of a similar protocol at the primary healthcare level.
